# Notes and News

**Published:** 1892-04-30

**Authors:** 


### NOTES AND NEWS.

COLLECTED AND COMMUNICATED.

An excellent form of charity has, we learn, been started in St. Louis by some lady inhabitants, in connection with the Protestant Hospital. This is a Dietry Dispensary for patients in their own homes. By means of this the sick poor are supplied with the nourishment considered necessary by written order of the doctor. Payment is required according to means, the very poor being supplied without charge. Considering the inability of the poor to cook for the sick-room, such charities should be of inestimable benefit.

Another useful charity emanates from the States. This takes the form of a society for the purpose of teaching street boys how to knit and mend, and even to proceed to more ambitious efforts in needle art. Those who have tried the experiment of occupying boys' hands in like useful manner will know the advantage of such employment for them.

In timely anticipation of the arrival into their midst of the International Congress of Hygiene of 1894, the sanitary authorities in Hungary are making a Lilliputian attempt to purge their Augean stables of their insanitary contents. They do not metaphorically turn on the rivers Alpheius and Peneius, and reach the *fons et origo malorum* by improving their defective drainage and water supply; but they make clean the outside of the platter by instituting a sensational campaign against fashionable ladies and their no less fashionable skirts; these, as they sweep the pavements, are charged with the crime of stirring up the germs of disease, and having stirred them up, of carrying them into the houses, and thus aiding and abetting the spread of tuberculosis and typhoid. The authorities should not rest content with sheering ladies of their elegant but dangerous plumage, the streets and pavements should daily be watered with some antiseptic solution, and before entering a house each individual should be sponged down with a similar solution, the boots which have been soiled by the contamination of the pavement being first left outside. Moreover, when practical, screens should be set up to restrain the vehemence of the winds and consequent widespread dissemination of pathological micro-organisms. In our own London hospitals one of the privileges on becoming a Sister of a ward is that of wearing a skirt with about four feet of trailing elegance behind; we now know of a surety how to explain the hitherto inexplicable spread of infectious diseases in these institutions.

The *Globe* points out that there are more ways than one of becoming celebrated, as in the case of Henry Irving, who "likes to tell" how, one day last summer in Dorsetshire, he passed a group of children, one of whom eyed him curiously. "Well, little girl," he said to her, "do you know me?" "Yes, sir," was the reply; "you are one of B——'s pills." She had seen his portrait in an advertisement "block."

The Metropolitan Drinking Fountains Association does a good work quietly and unostentatiously—a work both important and necessary. If our horses, dogs, and cattle could speak, they, at any rate, would pass a unanimous and enthusiastic vote of thanks to the society. A number of fountains and troughs for cattle, &c., have been erected during the past year, making up a total at the present time of nearly 700 fountains and about 750 troughs. The average annual amount paid for water by the society reaches the large sum of £1,500. We cordially commend the society to our readers.

The new out-patients' department of the Manchester Hospital for Consumption and Diseases of the Throat and Chest, which was opened last month, is nearly double the size of the former section. The new reception hall can accommodate 200 persons, and measures 40 ft. by 40 ft. It is lighted by an octagonal dome 30 ft. in height, which terminates in a turret. Round the hall are grouped nine consulting and examination rooms, each provided with double doors and windows for the exclusion of sound. There is a new dispensary in conjunction with the department. The ventilation, lighting, and heating have been carefully considered. The Chairman, Mr. Crossley, has presented a gas engine, which forms the motive power in the heating apparatus. The cost of the enlargement has exceeded £2,500. The designs were executed by Messrs. Pennington and Bridge.

Dr. Holman, Treasurer of the Royal Medical Benevolent College, writes: "It gives me great pleasure to inform you that Her Majesty has been pleased to contribute £25 towards the funds of the College. The *imprimatur* of Her Majesty's approval of the efforts of the Council to improve the condition of the College must surely stamp our scheme as being good. May I not hope that such an example may induce many others to aid us, and enable us to make a securely improved position for our pensioners; and, at the same time, to extend and to benefit our rapidly rising school? I would commend to the serious attention of your readers the idea of contributing a certain sum per annum for a term of years to this specific purpose." We heartily endorse this appeal; the institution is one that carries on a useful work in the face of many difficulties, though of late years the services it renders have been more fully recognised. No man devotes himself to a more self-denying and unselfish career than the general practitioner. Besides the unceasing call on his time and trouble, with frequently doubtful remuneration, he is required to keep up certain appearances, which renders saving a difficult matter, and causes that anxiety as to the future which may be one of the possible causes for the large mortality amongst the members of his profession. The Royal Medical Benevolent College has a large scope, therefore, for usefulness, in the provision of pensions to the disabled, and education for the children left unprovided for. We all owe a debt of gratitude to the medical profession, which we should welcome the opportunity of paying, by assisting the funds of this excellent institution.

